# The evolution trend of availability of China’s community-based care services and its impact on the cognitive function of elderly people: 2008-2018

**DOI:** 10.1186/s12939-021-01544-w

**Published:** 2021-09-08

**Authors:** Zhang Yue, Nan Xiang, Huwei Li, Erpeng Liu

**Affiliations:** 1 School of Public Administration, Zhongnan University of Economics and Law, Wuhan, 430073 China; 2 Institute of Income Distribution and Public Finance, Zhongnan University of Economics and Law, Room 130 in the Wenqin Building, No. 182 Nanhu Road, Donghu New Technology Development Zone, Wuhan, 430073 China; 3 Population and Health Research Centre, Zhongnan University of Economics and Law, Wuhan, 430073 China

**Keywords:** Community-based care services, Evolution trend, City-rural disparity, Chinese elderly people, Cognitive function

## Abstract

**Background:**

To address the challenge of the aging population, community-based care services (CBCS) have been developed rapidly in China as a new way of satisfying the needs of elderly people. Few studies have described the evolution trend of availability of CBCS in rural and urban areas and evaluated their effectiveness. This study aims to show the availability of China’s CBCS and further analyze the effect of the CBCS on the cognitive function of elderly people.

**Methods:**

Longitudinal analysis was performed using data from the 2008 to 2018 Chinese Longitudinal Healthy Longevity Survey (CLHLS). A total of 23937 observations from 8421 elderly people were included in the study. The Chinese version of the Mini-Mental State Examination (MMSE) was used to assess cognitive function. We aggregated similar CBCS to generate three binary variable categories (daily life support, emotional comfort and entertainment services, medical support and health services) indicating the availability of CBCS (1 = yes, 0 = no). Multilevel growth models were employed to estimate the association between CBCS and cognitive function while adjusting for many demographic and socioeconomic characteristics.

**Results:**

The availability of CBCS increased a lot from 2008 to 2018 in China. Although the availability of CBCS in urban areas was higher than that in rural areas in 2008, by 2018 the gap narrowed significantly. Emotional comfort and entertainment services (B = 0.331, 95% CI = 0.090 to 0.572) and medical support and health services (B = 1.041, 95% CI = 0.854 to 1.228) were significantly and positively associated with cognitive function after adjusting for the covariates.

**Conclusion:**

There was a significant increase in the availability of CBCS from 2008 to 2018 in China. This study sheds light on the positive correlation between CBCS and cognitive function among Chinese elderly individuals. The results suggest that policymakers should pay more attention to the development of CBCS and the equity of the supply of CBCS in urban and rural areas.

## Introduction

China is aging at an unprecedented rate, which raises great concern for public health. According to the latest statistics of the Chinese National Bureau of Statistics, by the end of 2020, the number of elderly people over the age of 60 nearly reached 264 million, accounting for 18.7% of the total population [1]. It is predicted that by 2050, the number of Chinese elderly people aged 60 years and over will reach a peak of 488 million, representing 35.6% of the total population [2]. At the same time, China is an unhealthy aging society. It is estimated that the disabled elderly population in China will increase from 27.113 million in 2020 to 65.514 million in 2050, and the total disability rate will increase from 10.8% in 2020 to 13.7% in 2050 [3]. The survey data from National Health Commission of the People’s Republic of China showed that more than 180 million Chinese elderly people suffer from chronic diseases, and it is common for the elderly to suffer from multiple chronic diseases [4]. Another survey shows that among the elderly people in China, 38.1% of them need visiting nursing service, 12.1% of them need housekeeping service, 11.3% of them need rehabilitation nursing service, 10.6% of them need psychological counseling service, 10.3% of them need health education service, 9.4% of them need daily care service [5]. With the increase in aging population and life expectancy, the number of elderly individuals with cognitive impairment is also growing dramatically in China [6]. Major cognitive impairment is clinically diagnosed as dementia [7]. The number of patients with dementia in China is estimated to be 10-11 million individuals aged 60 years and older or 9-10 million patients aged 65 years and older, and more than 60% of patients with dementia have Alzheimer’s disease [8]. Exploring the determinants of the cognitive function of elderly people and formulating appropriate intervention policies should be one of the most important tasks for China to address the challenge of the aging population.

China is a country that emphasizes filial piety [9]. Thus, family members have always played an important role in meeting the needs of their parents in terms of financial support, daily care, and spiritual comfort [10]. However, since the one-child policy was implemented in the 1970s, the family structure gradually transformed from traditional extended family to nuclear family [11]. During the process of urbanization and industrialization, increased population mobility has led to changes in parents’ and children’s co-residence models. As a result, the important role that the family plays in supporting the elderly population have gradually diminished [10, 12–14]. Considering this situation, the Chinese government began to develop CBCS (community-based care services). In 2008, the Office of the National Working Committee on Aging formulated the document “Opinions on Comprehensively Promoting Home Care Services”, which emphasized that the government and society should aim to provide CBCS reliant on community platforms, such as housekeeping services, rehabilitation care and spiritual comfort services for elderly individuals [15]. Enhancing the basic role of communities in meeting the various needs of elderly individuals has become the most important principle in the construction of China’s aging care system.

During the past 10 years, CBCS, as an important supplement to traditional family care services, have received increasing attention in the context of a rapidly aging population [16]. According to incomplete statistics, since 2010, the China central government has issued nearly 22 documents to promote the development of the CBCS. By the end of 2019, communities with care services and facilities accounted for 9.95% [17], and CBCS have progressed tremendously in China.

Under the conditions of relatively limited support resources for elderly individuals, it is more convenient and satisfying for Chinese elderly individuals to receive support from CBCS in their own home than living in a nursing home [18]. Living in private nursing homes is very expensive, and the majority of the elderly in China cannot afford the cost of private nursing homes [19, 20]. The elderly with better financial conditions (e.g., urban elderly, elderly with higher pensions) were more likely to live in private nursing homes [16, 21]. As for public nursing homes, they are mainly provided for the elderly in poverty without children. There were 1.623 million elderly people lived in public nursing homes in 2015, which only accounted for 0.73% of the total elderly population [22]. The data indicated that the elderly lived in nursing homes (including private and public nursing homes) only accounted for nearly 1%, and 99% elderly people lived in their own home [21]. Compared with the highly cost of private nursing homes and specific group targeted of public nursing homes, CBCS have advantages of convenience and inexpensive, and become the main choice for the Chinese elderly. However, most of the existing literature focuses on the supply and demand status of CBCS [23] and rarely explores whether CBCS can effectively improve the health of elderly individuals, especially delaying the cognitive decline of elderly people. A study showed that CBCS have the potential to support social participation for the growing number of adults aging at home [24]. Social participation can provide elderly individuals with daily cognitive stimulation, which is a protective factor for cognitive function [25]. CBCS are rich and diverse in China. In addition to providing daily life support for disabled elderly people, they include services such as organizing recreational activities for healthy elderly people and providing emotional comfort and entertainment services. A study based on 129 elderly participants found that cognitive training by visiting nursing services was more effective in improving the cognitive function of elderly individuals with cognitive dysfunction [26].

A large number of studies have focused on analyzing the effect of sociodemographic factors [27–29], socioeconomic factors [30–32], and physiological factors on the cognitive function of elderly individuals [33, 34]. However, few studies have examined the effects of CBCS on the cognitive function of elderly individuals. In terms of emotional comfort and entertainment services, some studies have shown that regular participation in cultural and entertainment activities can improve the cognitive function of elderly individuals and slow down the speed of cognitive decline [35, 36]. A few studies have also pointed out that emotional support can protect the cognitive function of elderly individuals [37], and elderly individuals who receive high-frequency emotional support have better comprehensive cognitive abilities [38]. Providing emotional comfort and entertainment services in the community can reduce psychological problems such as depression and anxiety in elderly people [39] and further protect their cognitive function [40, 41]. Daily life support, medical support and health services often have an indirect impact on the cognitive function of elderly individuals. Some studies have shown that negative psychological emotions, such as depression and anxiety, are related to cognitive impairment [40, 41]. The ability to meet the needs of daily life support, medical support, and health services is the key to maintaining the mental health of elderly individuals [42].

In the context of COVID-19, the possible variability of the virus and the uncertainty of its spread will make epidemic prevention and control the new normal for the foreseeable future. Under such circumstances, CBCS are of great significance in eliminating the adverse effects of the epidemic on the health of elderly people. Usually, elderly individuals receive social support, crisis assistance, and opportunities for social participation through family members or friends [43]. COVID-19 has aggravated the social isolation and loneliness of elderly individuals and has increased the risk of anxiety, loneliness, and depression in this population [44, 45]; which, in turn, has a negative effect on their cognitive function [40, 41, 46]. Some communities provide shopping services for elderly people, which effectively reduces the probability of infection. In addition, community-based medical support and health services and emotional comfort and entertainment services through a combination of online and offline methods can relieve anxiety and loneliness and improve cognitive function [44]. The COVID-19 epidemic highlights the importance of the community, and more attention should be given to the development of CBCS.

To the best of our knowledge, this is the first article to evaluate the evolution trend and the effects of CBCS on the cognitive function of elderly individuals in China. Compared with previous studies, this article mainly contributes to this field in the following aspects. First, the evolution trend of CBCS was clearly presented based on ten-year longitudinal data from 2008 to 2018. During this period, China experienced a rapidly aging population, and CBCS were developed from inception to widespread popularization. Second, we found clear evidence on the positive impact of CBCS on the cognitive function of elderly individuals in China and further subdivided CBCS into three categories, daily life support, emotional comfort and entertainment services, and medical support and health services, to analyze the effects of different types of CBCS on the cognitive function of elderly individuals. Unlike previous studies that only considered a certain type of CBCS, we explored the impact of CBCS on the cognitive function of elderly individuals from multiple dimensions to provide targeted solutions for improving the cognitive function of elderly individuals.

## Methods

### Data source

The data were derived from the Chinese Longitudinal Healthy Longevity Survey (CLHLS), a nationally representative survey jointly performed by the Center for Healthy Aging and Development Studies at Peking University and Duke University. This study aims to identify a set of social, behavioral, biological, and environmental risk factors that influence healthy longevity. The CLHLS was first conducted in 1998, followed by seven waves of surveys in 2000, 2002, 2005, 2008, 2011, 2014, and 2018 from 22 sample areas in 31 provincial administrative units. The sampling design of CLHLS adopted a multi-stage disproportionate and targeted random sampling method, taking into account the needs for a sample that was representative, methods that allowed for the collection of reliable data, and a field-work program that was feasible [47]. The population of the surveyed region accounts for 85.3% of the total population of the country, which can be regarded as a nationally representative sample [48]. The CLHLS study was approved by the Ethics Committee of Peking University (IRB00001052–13074). Detailed information about this survey, including specific objectives, organizational framework, study design, sample distribution, and contents of the data collected, is available in previous studies [49, 50].

The present study used data from the last four waves of datasets, including the 2008, 2011, 2014, and 2018 waves. We excluded individuals with missing values on any variable tested in this study. Respondents aged 60 and above were selected for analysis. The final analytic sample consisted of 23937 observations from 8421 respondents who completed, on average, approximately 3 waves of the survey (Mean = 2.84, SD = 0.81). There were 3328 respondents who participated in two waves, accounting for 40.36%, 2872 respondents who participated in three waves, accounting for 34.11%, and 2150 respondents who participated in four waves, accounting for 25.53%.

### Measures

#### Dependent variable: cognitive function

Cognitive function was used as the dependent variable in our research. Cognitive function was assessed by 24 items modified on the basis of the Mini-Mental State Examination [51], which has been proven to be reliable and effective with Chinese elderly individuals [50, 52, 53]. It contains a brief set of components to assess orientation, concentration, memory, and language, with total scores ranging from 0 to 30. Higher scores indicate better cognitive function.

#### Independent variables: community-based care services (CBCS)

Community-based care service (CBCS) has been advocated and became an increasingly important mode of care provision in China. “Community-based Care” is used in policy terms of China, and it is defined as professional care provided by government and social forces to the elderly within their locality [54–56]. Specifically speaking, community-based care services refer to a series of social services provided to the elderly people living in their own community [57], mainly including psychological support, regular medical examinations, the development of electronic medical records, legal counseling, grocery delivery, housekeeping, community kitchens, recreation centers, and mutual aid networks [19, 58]. In recent years, community-based care services (CBCS) have been developing rapidly in China to satisfy a wide range of situations and needs. Community-based care services have a long history in China, but they had been one of the most underdeveloped and vulnerable components of the Chinese health system [59]. Since the health care reforms implemented starting in 2008, the government has established more community medical centers and improved the capacity of existing centers to enhance disease prevention and health promotion [60].

Independent variables of the study consisted of daily life support, emotional support and entertainment services, and medical support and health services. In CLHLS, the participant was asked about the perceived availability of eight CBCS consisting of personal daily life care services, home visits, psychological support, daily shopping assistance, social and recreation activities, legal aid, health education, and neighboring relations. Respondents indicated “Yes” or “No” for the perceived availability of each service in their communities.

Considering that daily care services, home visits, psychological support, daily shopping, social and recreation activities, and health education are in greatest demand and the most common in the community, they have long-term effects on the cognitive function of elderly individuals. Therefore, this article focuses on analyzing the impact of these six services on the cognitive function of participants. In this study, we subdivide CBCS into three categories by similar type, namely, daily life support, emotional comfort and entertainment services, and medical support and health services. Specifically, daily life services include daily life care services and daily shopping assistance. Psychological support and social and recreation activities are grouped into emotional comfort and entertainment services. Home visits and health education are categorized as medical support and health services. Essentially, by grouping similar services together, the model explains the role that other services play but avoids the collinearity problems that arise when all variables are put into the same model [61]. Then, we set each CBCS as a binary variable depending on whether they were available in the participant’s community (yes = 1, no = 0). For example, daily life support represents the availability of personal daily care services and/or daily shopping services.

#### Covariates

All analyses presented here included a series of covariates associated with cognitive function as follows according to previous studies [27, 28, 31, 32]. First, sociodemographic characteristics, including gender, age, education, marital status and activities of daily living (ADL), were assessed. The Katz scale was adopted to evaluate activities of daily living (ADL), which included six items: bathing, dressing, bathroom use, indoor transferring, continence and eating [49, 50, 52]. Respondents were asked to rate their ability to engage in these ADLs using a three-point scale ranging from (1) not difficult at all, (2) slightly difficult, and (3) unable to do the task. The total scores ranged from 6 to 18, and higher scores indicated more limited functioning of the respondent. Second, family and social economic factors, including whether they are living alone, financial support sufficiently pays their daily expenses (yes = 1, no = 0), and their residence is in the city or the rural area (rural = 1, city = 0). Third, lifestyle included whether they had smoked/consumed alcohol/exercised in the past (yes = 1; no = 0).

### Statistical analysis

We first used descriptive analysis to describe the general characteristics of the participants and examine dynamic trends in the availability of CBCS and cognitive function among Chinese elderly individuals from 2008 to 2018 and the baseline characteristics of the study participants. Then, the availability of CBCS of participants between groups of different areas was reported, as well as the change in cognitive function of participants among groups with different sociodemographic characteristics. T tests and ANOVA tests were performed to compare the availability of CBCS and the change in cognitive function between different residence, gender, marital status, and education groups. Finally, considering that the four waves of longitudinal data might include time correlations within individuals, multilevel growth models were employed with maximum likelihood estimation (MLE), which is suitable for analyzing longitudinal data with repeated observations from the same individual over time and with panel attrition [62]. The multilevel growth models have more advantages than the traditional linear regression. First, it is appropriate for the nested nature of our data that individual observations over time are nested within persons. Second, it does not require all individuals be measured at each wave. Third, it allows us to estimate within-subject and between-subjects effects simultaneously [63]. The age variable was centered to make the intercept meaningful. All analyses were conducted using the statistical software Stata15 (Stata Corp, College Station, TX, USA).

## Results

The general characteristics of the participants at baseline are shown in Table 1. In this study, 3816 women (45.32%) and 4605 men (54.68%) were recruited. The average cognitive score of the participants was 23.085 at baseline. The average age and education of the participants were 82.585 and 2.305, respectively. In total, 3816 participants (45.32%) were married. A total of 6531 elderly people (77.51%) had sufficient financial support to pay daily expenses, while 1890 (22.44%) did not. A total of 5205 participants (61.81%) lived in rural areas, while 3216 (38.19%) participants lived in urban areas. Among participants, 2819 (33.48%) smoked, 2434 (28.90%) drank alcohol, 2784 (33.06%) exercised regularly in the past, and 1367 (16.23%) lived alone.

**Table 1:** Descriptive statistics of the study population (at baseline)

Variable	Measurement	N (%)/Mean (SD)
		(***n*** = 8421)
Cognitive function	Continuous measurement	23.085 (8.16)
Age	Continuous measurement	82.585 (11.04)
Gender	Male = 1	4605 (54.68)
	Female = 0	3816 (45.32)
Education (years)	Continuous measurement	2.368 (3.56)
Marital status	Have spouse = 1	3816 (45.32)
	Without spouse = 0	4605 (54.68)
ADL	Continuous measurement	6.342 (1.320)
Sufficient financial support for daily expenses	Yes = 1	6531 (77.56)
	No = 0	1890 (22.44)
Residence	Rural = 1	5205 (61.81)
	City = 0	3216 (38.19)
Smoked	Yes = 1	2819 (33.48)
	No = 0	5602 (66.52)
Consumed alcohol	Yes = 1	2434 (28.90)
	No = 0	5987 (71.10)
Exercised	Yes = 1	2784 (33.06)
	No = 0	5637 (66.94)
Living alone	Yes = 1	1367 (16.23)
	No = 0	7054 (83.77)
Daily life support	Yes = 1	528 (6.27)
	No = 0	7893 (93.73)
Emotional comfort and entertainment services	Yes = 1	1007 (11.96)
	No = 0	7414 (88.04)
Medical support and health services	Yes = 1	993 (11.79)
	No = 0	7428 (88.21)

The evolution trend of the availability of CBCS from 2008 to 2018 and the differences between urban and rural areas are displayed in Table 2. In general, the results show that there was a significant increase in the availability of CBCS among elderly people in China. However, the analysis by service type indicates some variance. In 2018, the proportion of elderly people reporting the availability of CBCS reached 14.66% (daily life support), 25.59% (emotional comfort and entertainment services), and 50.78% (medical support and health services). The results also showed that the gap between urban and rural areas is gradually narrowing in terms of the availability of daily life support, medical support and health services. In 2008, the availability of daily life support (*P* < 0.001), emotional comfort and entertainment services (*P* < 0.001), medical support and health services (*P* < 0.001) in urban areas was significantly higher than that in rural areas. In 2011, 2014 and 2018, there were significant differences in the availability of emotional comfort and entertainment services (*P* < 0.001) between urban and rural areas, while medical support and health services and daily life support did not differ significantly between urban and rural areas.

**Table 2:** The evolution trend of availability of CBCS and the differences between urban and rural areas (2008-2018)

		2008	***p- value***	2011	***p- value***	2014	***p- value***	2018	***p- value***
Daily life support (%)		6.27		8.91		12.05		14.66	
Residence (%)	urban	7.99	<0.001	9.73	0.006	11.81	0.567	15.87	0.067
	rural	5.21		8.01		12.34		13.12	
Emotional comfort and entertainment services (%)		11.96		17.76		23.21		25.59	
Residence (%)	urban	18.31	<0.001	23.09	<0.001	26.27	<0.001	29.37	<0.001
	rural	8.03		11.76		19.56		20.79	
Medical support and health services (%)		11.79		42.06		50.99		50.78	
Residence (%)	urban	16.82	<0.001	42.60	0.300	50.66	0.613	52.70	0.040
	rural	8.68		41.47		51.38		48.34	

Table 3 shows the changes in the cognitive function of the participants, as well as the differences in the cognitive function of the elderly participants by gender, marital status, residence, and education level. There were significant differences in the cognitive functions of elderly people in terms of gender, residence, marital status, and education level. Specifically, men and urban elderly individuals had better cognitive functions than women (*P* < 0.001) and rural elderly individuals (*P* < 0.001). Elderly individuals who are married have better cognitive functions than those who have no spouses (*P* < 0.001). Educated elderly individuals were less likely to experience cognitive decline than those illiterate counterparts (*P* < 0.001). There was no significant difference in the cognitive function of participants between urban and rural areas in 2014 and 2018.

**Table 3:** The changes in the cognitive function of participants and the differences among different genders, marital statuses, residences, and education levels (2008-2018)

		2008	***p- value***	2011	***p- value***	2014	***p- value***	2018	***p- value***
Gender	female	21.50	<0.001	20.50	<0.001	21.75	<0.001	22.16	<0.001
	male	25.00		24.52		25.21		25.23	
Residence	urban	23.76	<0.001	22.66	<0.001	23.52	0.167	23.95	0.022
	rural	22.67		21.95		23.19		23.15	
Marital status	married	25.86	<0.001	26.06	<0.001	26.10	<0.001	25.97	<0.001
	other	21.13		20.16		21.55		21.98	
Education	illiterate	20.91	<0.001	19.94	<0.001	21.22	<0.001	21.27	<0.001
	literate	25.96		25.46		25.76		25.76	

The results of multilevel growth models used to explore the association between CBCS and cognitive function in all participants are shown in Table 4. Before the two models in Table 4, we tested a null model, in which a single intercept is estimated and no predictor variables are included. The null model is mainly used to test whether the data is suitable for multilevel growth models. The within-and between-subjects variance components were 6.03 and 6.31 respectively, which indicates that it is suitable for multilevel growth models. Model 1 showed the sociodemographic factors that influenced cognitive function, while three types of CBCS were included in Model 2. Both the AIC (Akaike Information Criterion) and BIC (Bayesian Information Criterion) statistics show that the model with community-based care service fits better than Model1. The results indicated that emotional comfort and entertainment services (B = 0.331, 95% CI = 0.090 to 0.572) and medical support and health services (B = 1.041, 95% CI = 0.854 to 1.228) were significantly and positively associated with cognitive function after adjusting for the covariates. We also found that some sociodemographic characteristics, family and social economic factors, and lifestyle were significantly related to cognitive function. Age (B = -0.249, 95% CI = -0.261 to -0.238) had a significant negative impact on cognitive function, while education (B = 0.223, 95% CI = 0.190 to 0.256) and ADL (B = -1.412, 95% CI = -1.451 to -1.373) had a significant positive effect on cognitive function. Men had better cognitive function than women (B = 1.298, 95% CI = 1.026 to 1.570). Compared with living with family or living in a nursing home, living alone showed a significant positive correlation with cognitive function (B = 0.825, 95% CI = 0.578 to 1.073). Marriage had a protective function for elderly individuals (B = 0.802, 95% CI = 0.591 to 1.069). Having sufficient financial support for daily expenses (B = 0.931, 95% CI = 0.726 to 1.136), living in urban areas (B = -0.760, 95% CI = -0.937 to -0.583), and exercising regularly (B = 0.688, 95% CI = 0.464 to 0.912) were significantly and positively associated with cognitive function.

**Table 4:** The association between CBCS and cognitive function

Variable	Model 1			Model 2		
	B	S.E.	95% CI	B	S.E.	95% CI
Age	-0.244^***^	0.006	[-0.256, -0.233]	-0.249^***^	0.006	[-0.261, -0.238]
Gender	1.288^***^	0.139	[1.016, 1.561]	1.298^***^	0.139	[1.026, 1.570]
Education (years)	0.228^***^	0.017	[0.195, 0.261]	0.223^***^	0.017	[0.190, 0.256]
Marital status	0.830^***^	0.122	[0.591, 1.069]	0.802^***^	0.121	[0.564, 1.040]
ADL	-1.399^***^	0.020	[-1.438, -1.359]	-1.412^***^	0.020	[-1.451, -1.373]
Sufficient financial support for daily expenses	1.031^***^	0.105	[0.826, 1.236]	0.931^***^	0.104	[0.726, 1.136]
Residence	-0.875^***^	0.090	[-1.052, -0.698]	-0.760^***^	0.090	[-0.937, -0.583]
Smoked	0.017	0.137	[-0.251, 0.285]	-0.017	0.136	[-0.285, 0.250]
Consumed alcohol	-0.136	0.131	[-0.393, 0.122]	-0.151	0.131	[-0.408, 0.106]
Exercised	0.700^***^	0.114	[0.476, 0.924]	0.688^***^	0.114	[0.464, 0.912]
Living alone	0.825^***^	0.127	[0.577, 1.074]	0.825^***^	0.126	[0.578, 1.073]
Daily life support				0.137	0.152	[-0.161, 0.435]
Emotional comfort and entertainment services				0.331^**^	0.123	[0.090, 0.572]
Medical support and health services				1.041^***^	0.096	[0.854, 1.228]
**Goodness of fit**						
Log likelihood	-78510.841			-78416.077		
AIC	157051.7			156868.2		
BIC	157172.9			157013.7		
Number of observations	23937			23937		
Number of individuals	8421			8421		

*** *p* < 0.001, ** *p* < 0.01, * *p* < 0.05

## Discussion

In recent years, with the reduction in family size, the separation of intergenerational co-residence styles, and the weakening of filial piety culture, the traditional family intergenerational support pattern that child should take care of their parents by side has been changed [10, 13, 14]. Increasing numbers of elderly people live alone without receiving support from their children, such as daily care and spiritual comfort [64–66]. In response, the Chinese government vigorously developed the CBCS to compensate for the lack of family support. Although CBCS have achieved rapid development and considerable progress in China, little is known about their pervasiveness across relatively large countries or their associated impacts on the health of elderly individuals in China, especially cognitive function.

Longitudinal findings indicate the evolution trend of the availability of CBCS from 2008 to 2018 and the differences between urban and rural areas. In conclusion, the availability of CBCS for elderly people increased significantly in China from 2008 to 2018. However, some variance in the growth of CBCS was revealed through analysis by type of service. Medical support and health services were more accessible than other services, such as emotional comfort and entertainment services or daily life support reported by participants. Elderly people in urban areas were more likely to obtain CBCS than elderly people in rural areas. The results indicated that inequalities in primary care services still exist, but they are gradually decreasing. Therefore, it may be important to adopt additional strategies to further narrow the gap between the urban and rural areas in the availability of CBCS and to ensure that rural elderly people have equal access to CBCS.

The most important findings of this study were that emotional comfort and entertainment services and medical support and health services had a significant positive impact on the cognitive function of participants after adjusting for the covariates. The significant impact of emotional comfort and entertainment services on cognitive function may reflect the demands of spiritual comfort and interpersonal communication and activities among Chinese elderly individuals. Given the nature of kinship in Chinese society, family is the most important source for social contacts and social relationships [67, 68]. With the lack of family support from their children, elderly individuals who lose interpersonal interactions are more likely to be lonely. Community-based emotional comfort and entertainment services such as psychological support and recreational activities can help elderly individuals rebuild social connections and ease their psychological loneliness, which is beneficial to improve the cognitive function of elderly people [40, 41, 46]. For example, park opera groups and the Peking Opera groups are popular recreational activities among elderly individuals in China [69], creating platforms to communicate and interact, and satisfy the needs of spiritual comfort and social entertainment of elderly people.

It is worth noting that medical support and health services developed rapidly in China from 2008 to 2011. The data indicate that the proportion of elderly people reporting the availability of medical support and health services increased fourfold from 2008 to 2011 (in Table 2). The reason is that, starting in 2008, the Chinese government committed to developing community-based health care services. For example, in China’s “New Health Care Reform Plan” of 2009, the development of community-based health services was highlighted as one of the five key components of the government’s short-term targets for 2009 to 2011. Interventions targeting community health organizations, such as more subsidies from the government, increased investment in infrastructure, and well-trained staff, have been implemented rapidly since 2009 [57, 70]. These policies and measures have promoted the development of CBCS, which facilitates people’s access to health care and potentially improves health status, including cognitive function, among elderly individuals in China.

More work is needed to promote the development of CBCS to achieve wider availability. Xu et al. (2005) found that services provided at community centers varied greatly in quality and quantity, and there was generally no financial support from the government [69]. In addition, Wang (2011) pointed out that low willingness and the simplicity of activity types are two main problems of elderly people’s social involvement [71]. It is of great importance to further improve the accessibility and efficiency of CBCS to cope with the aging of the population in the future. What’s more, the caregivers in communities play an important role in CBCS. Policymakers’ attention should be paid on how to improve the social welfare of the caregivers. In order to promote the high-quality development of CBCS, the government must establish a social support system for caregivers who take care of the elderly in communities.

Nevertheless, the results should be interpreted cautiously for several limitations. First, the survey data provided information about the availability of CBCS but did not indicate whether the respondents used them. It has been proven that males who received fewer hours but more types of CBCS had significantly better cognitive performance [72]. Subject to the limitation of data, we only discussed the availability of CBCS. Since CLHLS is a face-to-face questionnaire, the elderly’s answers to CBCS-related questions are only a subjective perception. Thus, the utilization rate and service quality of CBCS might be explored in future study. Second, considering that the oldest-old individuals accounted for a relatively high proportion in this study, the results may be affected by the selective effect of death. Because the health status of the surviving oldest-old is generally better, the cognitive function score of participants may be higher overall. Thirdly, the limitation of multilevel growth model is that it ignores the heterogeneity of the groups, which needs to be solved by introducing the categorical latent variables in future studies.

## Conclusions

In conclusion, this study described the evolution trend in the availability of CBCS, discussed the difference between urban and rural areas, and provided evidence that CBCS are important factors to protect the cognitive function of elderly individuals while adjusting for many demographic and socioeconomic characteristics. There has been a significant increase in the availability of CBCS from 2008 to 2018, but the availability varies by the type of CBCS. Although there are still differences in CBCS between urban and rural areas, this difference has gradually decreased with the policy of equalization of CBCS in urban and rural areas being driven by the Chinese government. Emotional comfort and entertainment services, medical support and health services had significant positive impacts on cognitive function. The correlation between cognitive function and CBCS suggests that more targeted interventions should be undertaken to strengthen existing CBCS, especially daily life support. This result provides evidence about the availability and trend of CBCS for policy makers. In general, considering that less than ten percent of urban and rural communities supply relatively comprehensive care services, providing diversity and high-quality care services is still the key point of building an effective and sustainable aging care system in China.

## Data Availability

CLHLS data are available at https://opendata.pku.edu.cn/dataverse/CHADS (requiring a simple application).

## Abbreviations

ADL: Activities of Daily Living
AIC: Akaike Information Criterion
BIC: Bayesian Information Criterion
CBCS: Community-Based Care Services
CLHLS: Chinese Longitudinal Health Longevity Survey
MMSE: Mini-Mental State Examination

## References

[1] National Bureau of Statistics: Communiqué of China’s 7th national population census. Available from:http://www.stats.gov.cn/tjsj/zxfb/202105/t20210510_1817176.html. [cited 11 May 2021].

[2] United Nations. World population prospects: The 2012 revision. Available from: http://esa.un.org/unpd/wpp/Excel-Data/population.html. [cited 6 Feb 2021].

[3] Liao SH, Wang GZ (2021). Prevalence, changing trend of the elderly disability in China. Chin J Popul Sci.

[4] National Health Commission of the People’s Republic of China: Healthy China Action: health promotion action for the elderly. 29 Jul 2019. Available from: http://www.nhc.gov.cn/xwzb/webcontroller.do?titleSeq=11182&gecstype=1.

[5] Dang JW, Wei YY, Liu NN (2018). Survey on the living conditions of China’s urban and rural elderly.

[6] Alzheimer’s Disease International. The global impact of dementia: An analysis of prevalence, incidence, cost and trends. Available from: https://www.alzint.org/resource/world-alzheimer-report-2015/. [cited 6 Feb 2021].

[7] Julie H, Mary G (2014). Dementia and cognitive impairment: epidemiology, diagnosis, and treatment. Clin Geriatr Med.

[8] Jia LF, Quan MN, Fu Y (2020). Dementia in China: Epidemiology, clinical management, and research advances. Lancet Neurol.

[9] Ng ACY, Phillips DR, Lee WK (2002). Persistence and challenges to filial piety and informal support of older persons in a modern Chinese society: A case study in Tuen Mun, Hong Kong. J Aging Stud.

[10] Feng ZL, Glinskaya E, Chen HT, Gong S, Qiu Y, Xu JM, Yip WN (2020). Long-term care system for older adults in China: policy landscape, challenges, and future prospects. Lancet..

[11] Song L, Li S, Feldman MW (2008). Out-migration of young adults and gender division of intergenerational support in rural China. Res Aging.

[12] Yang JH, He ZH (2014). Continuity or change? Chinese family in transitional era. Popul Res.

[13] Giles J, Wang DW, Zhao CB (2010). Can China's rural elderly count on support from adult children? Implications of rural-to-urban migration. J Popul Ageing.

[14] Guo M, Aranda MP, Silverstein M (2009). The impact of out-migration on the inter-generational support and psychological wellbeing of older adults in rural China. Ageing Soc.

[15] The Central People’s Government of the People’s Republic of China. Opinions on comprehensively promote home-based care services. Available from: http://www.gov.cn/zwgk/2008-02/25/content_899738.htm. [cited 7 Feb 2021].

[16] Ge AL, Feng ZL (2018). Policy choice of China’s aged care: Building an efficient and sustainable aged care system in China.

[17] Mnistry of Civil Affair of the Republic of China. Statistical bulletin on the development of civil affairs 2019. Available from: http://www.mca.gov.cn/article/sj/tjgb/202009/20200900029333.shtml. [cited 7 Feb 2021].

[18] WHO. China country assessment report on ageing and health. Available from: https://apps.who.int/iris/bitstream/handle/10665/194271/9789245509318-chi.pdf?sequence=5. [cited 7 Feb 2021].

[19] Xu Q, Chow JC (2011). Exploring the community-based service delivery model: Elderly care in China. Int Soc Work.

[20] Feng TY, Mao DD (2020). How to promote to inclusive development of community home-based services for the elderly? A perspective of theoretical analysis. Chin Soc Secur Rev.

[21] Mu GZ (2012). The dilemma and solution of the development of institutional care in China. J Central China Normal Univ Human Soc Sci.

[22] Ministry of Civil Affairs of the People’s Republic of China. Statistical bulletin on social service development in 2015. Available from: http://www.mca.gov.cn/article/sj/tjgb/201607/20160715001136.shtml. [cited 11 July 2021]

[23] Peng CM, Burr JA, Kim K, Lu N (2020). Home and community-based service utilization among older adults in urban China: The role of social capital. J Gerontol Soc Work.

[24] Siette J, Berry H, Jorgensen M, Brett L, Georgiou A, McClean T, et al. Social participation among older adults receiving community care services. J Appl Gerontol. 2020;0733464820938973. 10.1177/0733464820938973. 10.1177/0733464820938973 32727252

[25] Choi MK (2020). Association between social participation and cognitive function among community-dwelling older adults living alone: Analysis of a nationally representative survey. Int J Nurs Pract.

[26] Park MH, Kwon DY, Seo WK, Lim KS, Song MS (2009). The effects of cognitive training on community-dwelling elderly Koreans. J Psychiatr Ment Health Nurs.

[27] Hayden KM, Reed BR, Manly JJ, Tommet D, Pietrzak RH, Chelune GJ, Yang FM, Revell AJ, Bennett DA, Jones RN (2011). Cognitive decline in the elderly:an analysis of population heterogeneity. Age Ageing.

[28] Yaffe K, Peltz CB, Ewing SK, Mcculloch CE, Cummings SR, Cauley JA, Hillier TA, Ensrud KE (2016). Long-term cognitive trajectories and mortality in older women. J. Gerontol Ser A-Biol Sci Med Sci.

[29] Van Gelder BM, Tijhuis M, Kalmijn S, Giampaoli S, Nissinen A, Kromhout D (2006). Marital status and living situation during a 5-year period are associated with a subsequent 10-year cognitive decline in older men: the FINE study. J Gerontol Ser B-Psychol Sci Soc Sci.

[30] Dartigues JF, Gagnon M, Letenneur L, Barberger-Gateau P, Commenges D, Evaldre M, Salamon R (1992). Principal lifetime occupation and cognitive impairment in a French elderly cohort(paquid). Am J Epidemiol.

[31] Andel R, Vigen C, Mack WJ, Clark LJ, Gatz M (2006). The effect of education and occupational complexity on rate of cognitive decline in alzheimer’s patients. J Int Neuropsychol Soc.

[32] Lee S, Kawachi I, Berkman LF, Grodstein F (2003). Education, other socioeconomic indicators, and cognitive function. Am J Epidemiol.

[33] Falck RS, Best JR, Davis JC, Eng JJ, Middleton LE, Hall PA, Liu-Ambrose T (2019). Sleep and cognitive function in chronic stroke: a comparative cross-sectional study. Sleep.

[34] Wang H, Fang C, Cai L, Dong B, Deng J (2016). Chronic kidney disease and cognitive impairment among the very old in China. Aging Clin Exp Res.

[35] Hughes TF, Flatt JD, Fu B, Chang CCH, Ganguli M (2013). Engagement in social activities and progression from mild to severe cognitive impairment: The MYHAT study. Int Psychogeriatr.

[36] Andel R, Finkel D, Pedersen NL (2015). Effects of preretirement work complexity and postretirement leisure activity on cognitive aging. J Gerontol Ser B-Psychol Sci Soc Sci.

[37] Iwasa H, Yoshida Y, Kai I, Suzuki T, Kim H, Yoshida H (2012). Leisure activities and cognitive function in elderly community-dwelling individuals in Japan: A 5-year prospective cohort study. J Psychosom Res.

[38] Seeman TE, Lusignolo TM, Albert M, Berkman L (2001). Social relationships, social support, and patterns of cognitive aging in healthy, high-functioning older adults: MacArthur studies of successful aging. Health Psychol.

[39] He ML, Long CL, Guo ZH (2013). Effect of group psychological intervention on anxiety and depression among empty nesters. Chin J Nurs.

[40] Donovan NJ, Wu Q, Rentz DM, Sperling RA, Marshall GA, Glymour MM (2017). Loneliness, depression and cognitive function in older U.S. adults. Int J Geriatr Psychiatry.

[41] Kassem AM, Ganguli M, Yaffe K, Hanlon JT, Lopez OL, Wilson JW, Cauley JA (2017). Osteoporotic Fractures in Men (MrOS) Study Research Group. Anxiety symptoms and risk of cognitive decline in older community-dwelling men. Int Psychogeriatr.

[42] Cai YY, Huang WY, Yang JY (2011). Influence of social support on incidence of MCI and its subtypes among the elderly. Chin J Public Health.

[43] Zhang WJ, Liu RP (2018). The status quo of the social isolation of the elderly in Chinese cities and the analysis of its influencing factors: based on the comparison between the migrant and non-migrated elderly. World Surv Res.

[44] Sepúlveda-Loyola W, Rodríguez-Sánchez I, Pérez-Rodríguez P, Ganz F, Torralba R, Oliveira DV, Rodríguez-Mañas L (2020). Impact of social isolation due to COVID-19 on health in older people: Mental and physical effects and recommendations. J Nutr Health Aging.

[45] Du P, An RX (2021). Impacts and enlightenments of COVID-19 on health services for the elderly. J Hebei Univ (Philos Soc Sci).

[46] Wei J, Ying M, Xie L, Chandrasekar EK, Lu H, Wang T, Li C (2019). Late-life depression and cognitive function among older adults in the U.S.: The National Health and Nutrition Examination Survey, 2011-2014. J Psychiatr Res.

[47] Zheng ZZ (2020). Twenty years’ follow-up on elder people’s health and quality of life. China Popul Dev Stud.

[48] Goodkind D, Poston DL (2009). Healthy longevity in China: demographic, socioeconomic, and psychological dimensions. Popul Stud-J Demogr.

[49] Feng Y, Liu EP, Yue Z, Zhang QL, Han TK (2019). The evolutionary trends of health behaviors in Chinese elderly and the influencing factors of these trends: 2005-2014. Int J Environ Res Public Health.

[50] Zeng Y, Feng QS, Hesketh T, Christensen K, Vaupel JW (2017). Survival, disabilities in activities of daily living, and physical and cognitive functioning among the oldest-old in China: A cohort study. Lancet..

[51] Folstein MF, Folstein SE, Mchugh PR (1978). Mini-Mental State: A practical method for grading the cognitive state of patients for the clinician. J Psychiatr Res.

[52] Feng QS, Zhu HY, Zhen ZH, Gu DA (2016). Self-rated health, interviewer-rated health, and their predictive powers on mortality in old age. J Gerontol Ser B-Psychol Sci Soc Sci.

[53] Deng QW, Liu WB (2021). Inequalities in cognitive impairment among older adults in China and the associated social determinants: a decomposition approach. Int J Equity Health.

[54] Wang C (2013). Reflections on the planning for comprehensive, long-term, and cost efficient community-based aging service facility system. City Plann Rev.

[55] Zhou J, Walker A (2016). The need for community care among older people in China. Ageing Soc.

[56] Zhu X (2017). Study on stratification characteristics and affecting factors of pension service demand of the old people in affordable housing community: taking Guangzhou as an example. Archit J.

[57] General Office of the State Council of the People’s Republic of China: The General Office of the State Council’ notice on printing and distributing the construction plan for the social elderly service system (2011-2015). 16 Dec 2011. Available from: http://www.gov.cn/xxgk/pub/govpublic/mrlm/201112/t20111227_64699.html.

[58] Wu B, Carter MW, Goins RT, Cheng C (2005). Emerging services for community-based long-term care in urban China: A systematic analysis of Shanghai’s community-based agencies. J Aging Soc Policy.

[59] Wang HH, Gusmano MK, Cao Q (2011). An evaluation of the policy on community health organizations in China: Will the priority of new healthcare reform in China be a success?. Health Policy.

[60] Chen Z (2009). Launch of the health-care reform plan in China. Lancet..

[61] Zhang YK, Yeager VA, Hou ST (2018). The impact of community-based supports and services on quality of life among the elderly in China: A longitudinal study. J Appl Gerontol.

[62] Singer JD, Willett JB (2003). Applied longitudinal data analysis: Modeling change and event occurrence.

[63] Lucas RE, Clark AE, Georgellis Y, Diener E (2003). Reexamining adaptation and the set point model of happiness: reactions to changes in marital status. J Pers Soc Psychol.

[64] Wang CW, Chan CL, Yip PS (2014). Suicide rates in China from 2002 to 2011: An update. Soc Psychiatry Psychiatr Epidemiol.

[65] Wu ZQ, Sun L, Sun YH, Zhang XJ, Tao FB, Cui GH (2010). Correlation between loneliness and social relationship among empty nest elderly in Anhui rural area, China. Aging Ment Health.

[66] Xie LQ, Zhang JP, Peng F, Jiao NN (2010). Prevalence and related influencing factors of depressive symptoms for empty-nest elderly living in the rural area of YongZhou, China. Arch Gerontol Geriatr.

[67] Chen Y, Hicks A, While AE (2014). Loneliness and social support of older people in China: A systematic literature review. Health Soc Care Community.

[68] Cheng ST, Lee CK, Chan AC, Leung EM, Lee JJ (2009). Social network types and subjective wellbeing in Chinese older adults. J Gerontol Ser B-Psychol Sci Soc Sci.

[69] Xu Q, Gao J, Yan MC (2005). Community centers in urban China: Context, development, and limitations. J Community Pract.

[70] Yip WCM, Hsiao WC, Chen W, Hu S, Ma J, Maynard A (2012). Early appraisal of China’s huge and complex health-care reforms. Lancet..

[71] Wang L (2011). A review of the theories, empirical evidence and policy research of the social involvement of elderly people in China. Popul Dev.

[72] Siette J, Georgiou A, Brayne C, Westbrook JI (2020). Social networks and cognitive function in older adults receiving home- and community -based aged care. Arch Gerontol Geriatr.

